# Supplementary material for: Genetic load makes cancer cells more sensitive to common drugs: evidence from Cancer Cell Line Encyclopedia
Source: Sci Rep. 2017 May 16;7:1938. doi: 10.1038/s41598-017-02178-1 (PMC5434051; doi:10.1038/s41598-017-02178-1)
Supplement: Supplementary file 1 — Supplementary Figures and Tables [file 41598_2017_2178_MOESM1_ESM.pdf]

Genetic load makes cancer cells more sensitive to common drugs: evidence from Cancer Cell Line Encyclopedia

Ana B. Pavel*[1] and Kirill S. Korolev*[1, 2]

[1]Graduate Program in Bioinformatics, Boston University, 44 Cummington Mall, Boston, MA 02215, USA; [2]Department of Physics, Boston University, 590 Commonwealth Ave, Boston, MA 02215, USA; e-mail: anapavel@bu.edu, korolev@bu.edu; *corresponding authors

Supplementary Figures and Tables

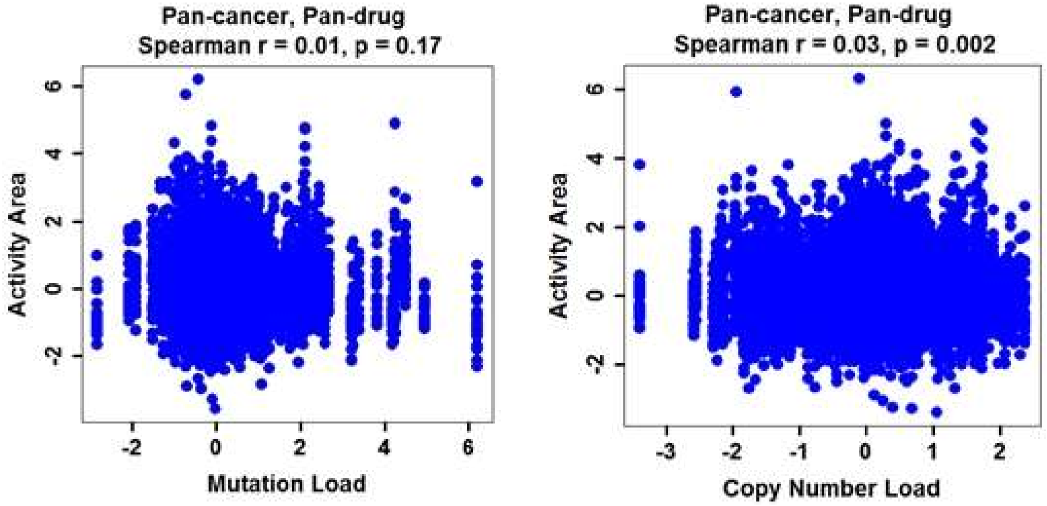

Supplementary Figure 1. Pan-data analysis for the mutation load (left) and the copy number load (right).

Supplementary Table 1. Twenty CCLE tissue types analyzed in this work.

TISSUE TYPE	Number of cell lines with profiled copy number data (1017)	Number of cell lines with profiled point mutation data (888)
HAEMATOPOIETIC AND LYMPHOID TISSUE	188	165
LUNG	185	172
CENTRAL NERVOUS SYSTEM	68	44
SKIN	61	53
BREAST	59	51
LARGE INTESTINE	59	56
OVARY	53	47
PANCREAS	44	37
STOMACH	39	34
KIDNEY	36	22
UPPER AERODIGESTIVE TRACT	31	31
ENDOMETRIUM	28	27
BONE	28	22
LIVER	27	24
OESOPHAGUS	27	25
URINARY TRACT	24	24
SOFT TISSUE	21	18
AUTONOMIC GANGLIA	17	15
THYROID	12	11
PLEURA	10	10

Supplementary Table 2. Significant associations for tissue-drug combinations with exclusion of tested drug-tissue combination from pan cancer and pan drug analyses. In the analysis of drug-tissue combinations, we preselected drugs and tissues based on the association with genetic load. Thus, some of the data was used twice: once in the selection step and once in the correlation test. Overall, the number of cell lines used twice was typically less than 10%, and, in many cases, pre-selection was based on a different measure of genetic load than the correlation test, i.e. on a different data. Nevertheless, we modified the analysis to ensure that statistical significance is not affected. We screened all tissue-drug combinations, not just those from Tables 2 and 3. For each combination, we first tested whether the tissue and the drug are associated with genetic load when the data from the combination is excluded. The combination was considered further only if pan-drug and pan-cancer analyses (with exclusion) showed positive association with the genetic load (Spearman FDR less than 0.1). For each combination passing this test, we computed the correlation between the Activity Area and the genetic load and performed FDR calculation based on the total number of combinations passing the test for each load.

Genetic load	Tissue type	Drug	Spearman		Pearson		No. cells
			ρ	FDR	r	FDR	
Point mutation load	LUNG	Lapatinib	0.39	0.08	0.55	0.02	15
Copy number load	ENDOMETRIUM	Lapatinib	0.69	0.01	0.58	0.04	20
	LIVER	Irinotecan	0.83	0.01	0.79	0.03	11
	ENDOMETRIUM	Erlotinib	0.64	0.06	0.47	0.12	20
Combined load	ENDOMETRIUM	Lapatinib	0.69	10^{-3}	0.58	0.01	20
	LIVER	Lapatinib	0.69	0.01	0.68	0.01	15
	ENDOMETRIUM	Erlotinib	0.6	0.01	0.47	0.03	20
	LIVER	Nilotinib	0.65	0.02	0.84	0.01	9
	STOMACH	TKI258	0.52	0.03	0.48	0.05	15
	LIVER	Topotecan	0.75	0.08	0.57	0.03	15

Supplementary Table 3. Associations between cancer pathways and point mutation load.

PATHWAY	Spearman		Pearson	
	ρ	FDR	r	FDR
REACTOME_APOPTOTIC_CLEAVAGE_OF_CELL_ADHESION_PROTEINS	0.25	10^{-7}	0.27	10^{-9}
ST_JNK_MAPK_PATHWAY	-0.24	10^{-7}	-0.13	0.01
REACTOME_GPCR_DOWNSTREAM_SIGNALING	-0.23	10^{-6}	-0.26	10^{-8}
PID_P38_ALPHA_BETA_PATHWAY	-0.21	10^{-5}	-0.15	10^{-3}
KEGG_MAPK_SIGNALING_PATHWAY	-0.21	10^{-5}	-0.12	0.01
REACTOME_ACTIVATION_OF_ATR_IN_RESPONSE_TO_REPLICATION_STRESS	0.19	10^{-4}	0.07	0.18
REACTOME_CELL_CYCLE_CHECKPOINTS	0.19	10^{-4}	0.11	0.02
REACTOME_SIGNALING_BY_GPCR	-0.19	10^{-4}	-0.26	10^{-9}
REACTOME_REGULATION_OF_INSULIN_LIKE_GROWTH_FACTOR_IGF_ACTIVITY_BY_INSULIN_LIKE_GROWTH_FACTOR_BINDING_PROTEINS_IGFBPS	-0.19	10^{-4}	-0.13	0.01
REACTOME_G2_M_CHECKPOINTS	0.18	10^{-4}	0.07	0.18
PID_LYMPH_ANGIOGENESIS_PATHWAY	-0.15	10^{-3}	-0.17	10^{-4}
REACTOME_G2_M_DNA_DAMAGE_CHECKPOINT	0.13	0.01	0.05	0.38
REACTOME_P53_DEPENDENT_G1_DNA_DAMAGE_RESPONSE	0.12	0.01	0.12	0.01
REACTOME_P53_INDEPENDENT_G1_S_DNA_DAMAGE_CHECKPOINT	0.12	0.01	0.05	0.34
PID_E2F_PATHWAY	0.12	0.01	0.06	0.23
REACTOME_GROWTH_HORMONE_RECEPTOR_SIGNALING	-0.11	0.02	-0.07	0.21
KEGG_MTOR_SIGNALING_PATHWAY	-0.11	0.02	-0.14	10^{-3}
REACTOME_DNA_REPAIR	0.11	0.02	0.03	0.61
REACTOME_APOPTOTIC_EXECUTION_PHASE	0.11	0.02	0.17	10^{-4}
REACTOME_EXTRINSIC_PATHWAY_FOR_APOPTOSIS	-0.10	0.03	-0.02	0.71
PID_ERBB_NETWORK_PATHWAY	0.10	0.03	0.12	0.01
PID_ATM_PATHWAY	0.10	0.04	0.02	0.71
REACTOME_APOPTOTIC_CLEAVAGE_OF_CELLULAR_PROTEINS	0.09	0.04	0.12	0.01
KEGG_NOTCH_SIGNALING_PATHWAY	0.09	0.05	0.15	10^{-3}
REACTOME_NRAGE_SIGNALS_DEATH_THROUGH_JNK	-0.09	0.05	0.14	10^{-3}
REACTOME_SIGNALING_BY_HIPPO	0.08	0.07	0.05	0.33

Supplementary Table 4. Associations between cancer pathways and copy number load.

PATHWAY	Spearman		Pearson	
	ρ	FDR	r	FDR
PID_P38_ALPHA_BETA_DOWNSTREAM_PATHWAY	0.12	0.03	0.11	0.06
REACTOME_HOMOLOGOUS_RECOMBINATION_REPAIR_OF_REPLICATION_INDEPENDENT_DOUBLE_STRAND_BREAKS	0.12	0.03	0.12	0.06
REACTOME_DOUBLE_STRAND_BREAK_REPAIR	0.12	0.03	0.12	0.06
PID_E2F_PATHWAY	0.12	0.03	0.07	0.39
REACTOME_P53_INDEPENDENT_G1_S_DNA_DAMAGE_CHECKPOINT	0.11	0.04	0.09	0.14
PID_ATM_PATHWAY	0.11	0.04	0.10	0.07
REACTOME_DNA_REPAIR	0.10	0.09	0.09	0.14

Supplementary Table 5. Associations between epithelial growth factor receptors and other related genes and point mutation load.

GENE	Spearman		Pearson	
	ρ	FDR	r	FDR
TGFB1	-0.21	10^{-5}	-0.14	0.01
PML	-0.21	10^{-5}	-0.20	10^{-4}
ERBB3	0.18	10^{-4}	0.16	10^{-3}
SGK3	0.19	10^{-4}	0.08	0.16
SMAD3	-0.17	10^{-4}	-0.08	0.17
TGFB2	-0.17	10^{-4}	-0.11	0.03
MMP2	-0.17	10^{-4}	-0.12	0.02
MYLK	-0.16	10^{-3}	-0.13	0.01
PAK2	-0.16	10^{-3}	-0.12	0.03
FGFR4	0.15	10^{-3}	0.17	10^{-3}
JAG2	0.14	10^{-3}	0.14	0.01
BRCA1	0.13	0.01	0.05	0.45
FGFR1	-0.14	0.01	-0.04	0.60
VEGFC	-0.13	0.01	-0.15	10^{-3}
ELF4	-0.13	0.01	-0.10	0.07
PTEN	-0.11	0.04	-0.04	0.54
ERBB2	0.10	0.06	0.14	0.01
INHBA	-0.10	0.07	-0.09	0.12
